# Meetings of Societies

**Published:** 1935

**Authors:** 


### Meetings of Societies.

### Bristol Medico-Chirurgical Society

THE seventh meeting of the session was held on Wednesday, 10th April, at 8.30 p.m., in the Physiological Lecture Theatre of the University, when Mr. Rendle Short was in the Chair and fifty-three members were present.

Dr. A. D. Fraser and Dr. F. J. A. Mayes showed pathological specimens and skiagrams respectively.

The minutes of the preceding meeting were read and confirmed. The Hon. Treasurer asked that the proposal with regard to the joint subscription of husband and wife made at the Annual Meeting of the Society should be ratified. The President proposed this from the Chair, and it was carried unanimously.

Dr. Norman Burgess then read a short paper on "**Eczema**

**and Dermatitis**" (see p. 103) which was illustrated by numerous diagrams and plates, and a discussion followed in which the President, Dr. Nixon and Dr. Gee took part, and Dr. Burgess replied.

The President called on Dr. J. Innes Noble to read a short paper on "**Medical Treatment of Hyperthyroidism**" (see p. 113). The paper was discussed by the President, Dr. Carleton, Dr. Casson and Dr. Frank Bodman, and Dr. Noble replied.

---

THE last meeting of the session was held in the Physiological Lecture Theatre of the University on Wednesday, 8th May, 1935, at 8.30 p.m.

The President, Mr. Rendle Short, was in the Chair, and sixty-six members were present.

Dr. A. L. Taylor showed pathological specimens.

The minutes of the previous meeting were read and confirmed, and Dr. C. Corfield proposed that Messrs. C. J. Ryland & Co. should be re-appointed Auditors to the Society for the coming year, and this was seconded by Dr. Richard Clarke.

The President then called on Mr. Hey Groves to give an address on the treatment of fractures, entitled "**The Ten Commandments.**" The President congratulated Mr. Groves on his address, and a discussion followed in which Mr. Nicholson Lailey, Dr. Philip Martin, Mr. Paul, Mr. Pridie, Dr. Forrester-Brown and the President took part.

